# Anatomical distribution of *Toxoplasma gondii* in naturally and experimentally infected lambs

**DOI:** 10.1051/parasite/2022001

**Published:** 2022-02-01

**Authors:** Myriam Thomas, Dominique Aubert, Sandie Escotte-Binet, Benoît Durand, Céline Robert, Régine Geers, Annie Alliot, Guillaume Belbis, Isabelle Villena, Radu Blaga

**Affiliations:** 1 French Agency for Food, Environmental and Occupational Health and Safety (ANSES), INRAE, École Nationale Vétérinaire d’Alfort, UMR BIPAR, Animal Health Laboratory 14 rue Pierre et Marie Curie Maisons-Alfort 94700 France; 2 National Reference Centre on Toxoplasmosis, Toxoplasma Biological Reference Centre, CHU Reims and EA7510, SFR CAP-Santé, University of Reims Champagne-Ardenne USC EpiToxo Anses Reims 51095 France; 3 Epidemiology Unit, Animal Health Laboratory, French Agency for Food, Environmental and Occupational Health and Safety (ANSES), University Paris-Est 14 rue Pierre et Marie Curie Maisons-Alfort 94700 France; 4 École Nationale Vétérinaire d’Alfort 7 avenue du Général de Gaulle Maisons-Alfort 94700 France

**Keywords:** *Toxoplasma gondii*, Lambs, Anatomical distribution, Meat

## Abstract

Consumption of raw or undercooked meat containing *Toxoplasma gondii* tissue cysts is one of the main sources of infection for humans worldwide. Among the various species intended for human consumption, sheep appear to be a high risk for human infection. The present study focused on the detailed anatomical distribution of *Toxoplasma gondii* in naturally and experimentally infected lambs using fresh and frozen samples of various pieces of meat, from a public health perspective. The first objective was to rank the edible parts intended for human consumption according to the detectable parasite burden by real-time PCR targeting the 529-bp repeated element. The second objective was to evaluate the impact of freezing by comparing the detection efficiency of the quantitative PCR between fresh and frozen tissues, as imports of lamb carcasses/cuts may arrive frozen or chilled. The highest estimated parasite loads were observed in skeletal muscles, and more particularly in edible portions such as quadriceps femoris muscle, intercostal muscles, deltoid muscle and diaphragm, with a significant difference in detectable parasite burden between fresh and frozen samples (*p* < 0.0001) or natural and experimental infection (*p* < 0.0001). Thoracic and pelvic limbs (3278–1048 parasites/g muscle) were ranked at the top of the list. *Toxoplasma gondii* DNA was detected in all the edible parts of lamb studied. These results suggest that lamb meat represents a risk for consumers. Further investigations are needed in order to confirm these differences in larger numbers of animals and in different breeds.

## Abstract (fr)

La consommation de viande crue ou insuffisamment cuite contenant des kystes tissulaires de *Toxoplasma gondii* est l’une des principales sources d’infection pour l’homme dans le monde. Parmi les différentes espèces destinées à la consommation humaine, le mouton apparaît à haut risque d’infection humaine. La présente étude s’est concentrée sur une distribution anatomique détaillée de *Toxoplasma gondii* chez des agneaux infectés naturellement et expérimentalement à l’aide d’échantillons frais et congelés de divers morceaux de viande, du point de vue de la santé publique. Classer les parties comestibles destinées à la consommation humaine, selon la charge parasitaire détectable par une PCR en temps réel ciblant l’élément répété de 529 pb était un premier objectif. Un second objectif était d’évaluer l’impact de la congélation en comparant l’efficacité de détection de la PCR quantitative entre les tissus frais et congelés, car les importations de carcasses/coupes d’agneau peuvent arriver congelées ou réfrigérées. Les charges parasitaires estimées les plus élevées ont été observées dans les muscles squelettiques et plus particulièrement dans les parties comestibles telles que le quadriceps fémoral, les muscles intercostaux, le deltoïde et le diaphragme avec une différence significative de charge parasitaire détectable entre les échantillons frais et congelés (p < 0,0001) ou l’infection naturelle et expérimentale (p < 0,0001). Les membres thoraciques et pelviens (3278 à 1048 parasites/g de muscle) ont été classés en tête de liste. L’ADN de *T. gondii* a été détecté dans toutes les parties comestibles étudiées de l’agneau. Ces résultats suggèrent que l’agneau représente un risque pour les consommateurs. Des investigations supplémentaires doivent être effectuées afin de confirmer les différences mentionnées ci-dessus chez plus d’animaux et dans différentes races.

## Introduction

The obligate intracellular coccidian parasite*, Toxoplasma gondii,* is responsible for one of the most important zoonoses worldwide, toxoplasmosis, with more than one third of the global population being infected [46]. Experts from Food and Agriculture Organization of the United Nations (FAO) and World Health Organization (WHO) have ranked *T. gondii* fourth, out of 24 foodborne parasites (FBP) of global importance [6]. With a similar approach, the European Network for Foodborne Parasites (Cost Action Euro-FBP) has recently ranked *T. gondii* as the second most important FBP in Europe [7]. Transmission to humans occurs mainly via one of the following routes: a) ingestion of raw or undercooked meat containing tissue cysts with bradyzoites; b) ingestion of vegetables, fruits or water contaminated by sporulated oocysts from cat (definitive host) stools, or c) congenital transmission from mother to fetus by tachyzoites [26]. Consumption of raw or undercooked meat from infected warm-blooded animals is considered a major source of foodborne toxoplasmosis in humans worldwide [3, 4, 45]. In Europe, consumption of undercooked infected meat has been attributed to 30–63% of infections [10]. Pigs, sheep and goats are generally considered to be frequent carriers of *T. gondii* tissue cysts, rather than cattle and horses [45, 46]. In the United States, a case-control study found that raw ground beef, rare lamb or locally produced cured, dried or smoked meats are among the most important risk factors for foodborne toxoplasmosis [30]. In Norway and France, undercooked lamb was found to be a risk factor for human toxoplasmosis [1, 33]. However, the exact role of infected lamb in the epidemiology of toxoplasmosis in humans remains undetermined, since in Europe, lower seroprevalences have been observed in lambs than in adults [5, 20, 24, 33], with a strong correlation between indirect and direct methods of *T. gondii* detection [37, 38].

Several authors have previously found viable *T. gondii* parasites in tissues like the brain, heart or diaphragm in naturally or experimentally infected sheep [18, 20–22, 24, 32, 42].

However, there are very few data about the presence of viable parasites in various edible pieces of meat intended for human consumption. In France, live parasites have been detected in sheep carcasses and 1/3 were isolated from lambs (animals younger than 12 months of age) [24]. The present study aimed to gain knowledge on the detailed anatomical distribution of *T. gondii* DNA in experimentally and naturally infected lambs, in order to rank the tissues according to the detectable parasite burden and therefore the risk for *T. gondii* contamination in humans.

Moreover, this study aimed to evaluate the impact of freezing on the detection efficiency of *T. gondii* by qPCR assay, since meat is often shipped frozen, and in some cases, such as ovine meat, this freezing can last up to several months (e.g. ovine meat produced in New Zealand and shipped to Europe).

## Materials and methods

### Lambs and experimental design

Four 3-month-old lambs (Pré Alpes du Sud), *Toxoplasma gondii* seronegative, were bought from a sheep flock of the French National Institute of Agricultural Research (INRAE, UCEA, Jouy-en-Josas, France) and used for experimental infection. The absence of *T. gondii* immunoglobulin G (IgG) antibodies was assessed by a modified agglutination test (MAT) as described below.

Three 3-month-old lambs (Vendéen) were selected from a regular sheep flock, based on the MAT result (MAT titer > 1:25) and considered *T. gondii* naturally infected lambs. The sheep flock was part of an educational farm in Champignelles, France belonging to the French National Veterinary School of Alfort (ENVA).

#### Experimental infection

The experimental infection was approved by the local Ethics Committee for animal experiments of ANSES/ENVA/UPEC (registration number: 10-0006; permission number: 10/03/09-03) and was carried out at the Biomedical Research Center of ENVA (Maisons-Alfort, France). All lambs were handled in strict accordance with good clinical practices and all efforts were made to avoid suffering.

*Toxoplasma gondii* oocysts (ME49 strain, genotype II, aged 6 months) isolated as described by Dubey [19] were obtained as a courtesy of Dr. JP Dubey (US Department of Agriculture, Agricultural Research Service, Beltsville, MD, USA) and stored in a 2% H_2_SO_4_ solution at 4 °C until use. Three 3-month-old lambs (E1, E2 and E3, Pré Alpes du Sud) were infected by oral administration of 10^3^ sporulated oocysts in a final volume of 20 mL of phosphate-buffered saline (PBS). A fourth animal, used as negative control, received only PBS. The animals were monitored for 8 weeks post infection (p.i.), and their health and behaviour were examined daily. Rectal temperatures were recorded daily from day 3 p.i. until day 22 p.i. A febrile response was defined as a rectal temperature of 40 °C or higher for one or more days. Weights were recorded and blood samples were taken from the jugular vein daily from day 3 p.i. until day 22 p.i., and weekly from day 22 p.i. until the end of the experiment. The lambs were euthanized 8 weeks p.i. according to animal welfare rules (intravenous (IV) injection of T61 in a controlled manner, after sedation with acepromazine).

#### Natural infection

Natural infection was studied on the educational farm of Champignelles, ENVA.

After selection, the three 3-month-old lambs (N1, N2 and N3, Vendéen) were euthanized in the autopsy facilities of the ENVA in Champignelles, according to animal welfare rules (intravenous (IV) injection of T61 in a controlled manner, after sedation with acepromazine).

### Sampling protocol

Nine tissues (brain, heart, lung, intestine, tongue, diaphragm, quadriceps femoris muscle, intercostal muscles, and deltoid muscle samples) were sampled from each experimentally or naturally infected lamb. The nine tissues from both experimentally (E1, E2 and E3) and naturally (N1, N2 and N3) infected animals were processed immediately after collection and tested for the detection of viable *Toxoplasma gondii* tissue cysts by mouse bioassay, and *T. gondii* DNA by quantitative PCR, as described below.

In addition, 34 muscles from the head, neck, back, thorax, thoracic and pelvic limbs, abdomen and tail were collected from the three experimentally infected lambs (E1, E2 and E3: Table 1). The 34 muscles were stored at −20 °C until analysis. The detection of *T. gondii*-specific immunoglobulin G (IgG) was performed on meat juice samples by a modified agglutination test (MAT), and the presence of *T. gondii* DNA was determined by quantitative PCR as described below.

**Table 1:** Supplementary sampled muscles (*n* = 34): MAT titer for the collected meat juice and detectable parasite burden (arithmetic mean of *T. gondii* parasites per gram of muscle) of experimentally infected lambs (E1, E2, E3) ranked from high to low. The Latin terminology of the muscles and their anatomical region were extracted from the “Nomina Anatomica Veterinaria, 6th edition 2017”. NR: negative results (Ct value > 40).

Muscle	Anatomical region	Position to the trunk	Role for physiology	E1			E2			E3			Arithmetic mean
				MAT titer	g^a^	p/g^b^	MAT titer	g^a^	p/g^b^	MAT titer	g^a^	p/g^b^	Range [min–max]
1. *Musculus supraspinatus*	Thoracic limb	Proximal	Postural	24	18.08	8238.53	nmj	34.70	819.78	96	101.25	778.48	3278.93
													[778.48–8238.53]
2. *Musculus subscapularis*	Thoracic limb	Proximal	Postural	48	15.25	8152.61	nmj	23.95	721.30	48	36.02	442.78	3105.56
													[442.78–8152.61]
3. *Musculus triceps brachii*	Thoracic limb	Proximal	Postural	48	29.02	6139.34	nmj	57.10	1182.42	96	122.12	1409.50	2910.42
													[1182.42–6139.34]
4. *Musculus infraspinatus*	Thoracic limb	Proximal	Postural	96	14.90	6312.15	nmj	22.66	701.46	96	60.32	1443.25	2818.95
													[701.46–6312.15]
5. *Musculus biceps femoris*	Pelvic limb	Proximal	Post/Phasic	24	26.50	3150.34	nmj	13.45	722.18	nmj	82.33	2861.79	2244.77
													[722.18–3150.34]
6. *Musculus adductor*	Pelvic limb	Proximal	Phasic	96	20.70	4889.02	nmj	65.13	213.83	96	37.88	1395.68	2166.18
													[213.83–4889.02]
7. *Musculus psoas major*	Pelvic limb	Proximal	Postural	96	14.50	3350.45	nmj	38.10	2167.83	96	41.65	840.91	2119.73
													[840.91–3350.45]
8. *Musculus biceps brachii*	Thoracic limb	Proximal	Phasic	24	25.08	5050.62	96	22.82	364.66	96	37.24	397.76	1937.68
													[364.66–5050.62]
9. *Musculus extensor digitorum brevis*	Pelvic limb	Distal	Phasic	96	16.31	2675.82	nmj	39.81	1147.43	96	54.74	1087.01	1636.75
													[1087.01–2675.82]
10. *Musculus flexor digitorum superficialis*	Pelvic limb	Distal	Phasic	96	15.65	3297.36	nmj	33.11	639.47	96	30.14	277.94	1404.92
													[277.94–3297.36]
11. *Musculus gracilis*	Pelvic limb	Proximal	Phasic	96	10.05	3045.79	nmj	15.31	445.09	96	51.45	430.18	1307.02
													[430.18–3045.79]
12. *Musculus gastrocnemius*	Pelvic limb	Distal	Postural	96	11.35	2321.97	nmj	18.95	725.99	48	45.88	826.16	1291.37
													[725.99–2321.97]
13. *Musculus semimembranosus*	Pelvic limb	Proximal	Phasic	neg	20.10	1674.60	96	103.50	659.46	nmj	94.30	811.55	1048.54
													[659.46–1674.60]
14. *Musculus serratus ventralis thoracis*	Thorax	Ventral	Post/Phasic	48	12.05	2017.16	nmj	33.95	421.67	nmj	59.95	345.80	928.21
													[345.80–2017.16]
15. *Musculus longissimus*	Back	Proximal	Postural	24	17.10	1313.59	48	113.44	639.79	96	161.47	648.78	867.39
													[639.79–1313.59]
16. *Musculus obliquus externus abdominis*	Abdomen	Ventral	Postural	24	19.50	1678.38	nmj	25.13	79.99	96	81.06	400.35	719.57
													[79.99–1678.38]
17. *Musculus coccygeus*	Tail	Caudal	Phasic	nmj	8.18	1778.54	nmj	9.75	226.33	nmj	5.85	38.86	681.24
													[38.86–1778.54]
18. *Musculus pterygoideus*	Head	Cranial	Postural	neg	3.70	1965.47	nmj	4.95	49.19	96	11.87	13.49	676.05
													[13.49–1965.47]
19. *Musculus flexor digitorum brevis*	Thoracic limb	Distal	Postural	96	18.52	797.12	nmj	26.28	721.71	nmj	73.70	431.59	650.14
													[431.59–797.12]
20. *Musculus gluteus medius*	Pelvic limb	Proximal	Phasic	24	6.54	NR	96	13.88	627.36	96	85.20	1012.62	546.66
													[0.00–1012.62]
21. *Musculus pectineus*	Pelvic limb	Proximal	Phasic	96	9.22	1066.49	nmj	11.50	58.74	96	15.70	436.85	520.69
													[58.74–1066.49]
22. *Musculus semitendinosus*	Pelvic limb	Proximal	Post/Phasic	48	24.35	362.77	nmj	39.85	454.26	nmj	104.58	723.45	513.49
													[362.77–723.45]
23. *Musculus tensor fasciae latae*	Pelvic limb	Proximal	Phasic	neg	16.90	1098.69	nmj	8.70	90.28	nmj	17.20	130.62	439.86
													[90.28–1098.69]
24. *Musculus sternocephalicus Pars mandibularis*	Neck	Ventral	Phasic	nmj	3.30	8.16	nmj	15.38	774.90	48	23.98	498.24	427.10
													[8.16–774.90]
25. *Musculus pectoralis profundus*	Thorax	Ventral	Postural	48	5.65	141.87	nmj	48.90	37.86	nmj	71.41	1078.72	419.48
													[37.86–1078.72]
26. *Musculus masseter*	Head	Cranial	Postural	nmj	7.50	981.36	nmj	9.75	180.81	96	21.74	44.49	402.22
													[44.49–981.36]
27. *Musculus latissimus dorsi*	Back	Dorsal	Phasic	nmj	4.93	18.13	nmj	17.00	461.57	nmj	60.02	474.84	318.18
													[18.13–474.84]
28. *Musculus extensor digitorum lateralis*	Thoracic limb	Distal	Phasic	nmj	10.35	79.93	96	22.75	378.86	nmj	69.41	441.81	300.20
													[79.93–441.81]
29. *Musculus temporalis*	Head	Cranial	Postural	nmj	1.90	686.03	nmj	3.34	NR	nmj	4.51	80.17	255.40
													[0.00–686.03]
30. *Musculus trapezius*	Back	Dorsal	Phasic	nmj	4.10	2.44	nmj	7.25	635.63	96	32.10	104.70	247.59
													[2.44–635.63]
31. *Musculus brachiocephalicus*	Neck	Ventral	Phasic	neg	7.10	NR	nmj	12.90	58.96	96	49.95	552.54	203.83
													[0.00–552.54]
32. *Musculus sartorius*	Pelvic limb	Proximal	Phasic	nmj	1.00	5.82	nmj	6.52	97.12	nmj	29.30	133.89	78.94
													[5.82–133.89]
33. *Musculus buccinator*	Head	Cranial	Phasic	nmj	3.25	25.92	96	9.68	21.56	nmj	4.21	32.58	26.69
													[21.56–32.58]
34. *Musculus digastricus*	Head	Cranial	Phasic	nmj	1.00	NR	nmj	1.27	11.33	nmj	3.20	NR	3.78
													[0.00–11.33]

nmj: no meat juice collected. neg = no IgG detected.

a g: grams of muscle.

b p/g: calculated number of parasites per gram.

The number of *T. gondii* parasites was calculated by interpolating the average Ct values on the standard curve equivalent to 1 × 10^0^ to 1 × 10^3^ tachyzoites with tenfold serial dilutions. Estimated parasite numbers in muscles (detectable parasite burden) were expressed as parasite number/g lamb muscle. The range describes the lowest and highest estimated number of parasites within samples.

Sera from the three experimentally infected lambs (E1, E2 and E3) were obtained by centrifugation of the blood samples collected daily from day 3 p.i. until day 22 p.i., and weekly from day 22 p.i. until the end of the experiment. The sera were analyzed by a modified agglutination test (MAT) as described below.

### Modified agglutination test

Lamb sera (E1, E2 and E3) were analyzed by MAT for the detection of *T. gondii*-specific immunoglobulin G (IgG) using an antigen prepared from formalin-fixed whole RH [16] provided by the French National Reference Center for Toxoplasmosis in Reims, France [50]. Serum samples were diluted two-fold starting at a 1:6 dilution up to a 1:12,288 dilution.

Meat juice samples, collected from frozen muscles (34 muscles × 3 lambs E1, E2 and E3) after thawing, were screened at 1:6 dilution and diluted to 1:96 dilution.

### Trypsic digestion

The nine tissues (brain, heart, lung, intestine, tongue, diaphragm, quadriceps femoris muscle, intercostal muscles, and deltoid muscle samples) from both naturally and experimentally infected animals were minced in a blender within 24 h after sampling, and then divided into two parts of the same weight. One part was digested immediately and the other part was digested after storage at −20 °C for 2–3 weeks. The first (fresh) digested part was used for bioassay and quantitative PCR (qPCR) and the second (frozen) for qPCR only. The other 34 muscles from the experimentally infected lambs were also digested after several weeks of storage at −20 °C. Briefly, each tissue was incubated for 1.5 h at 37 °C with trypsin solution (trypsin at final concentration 0.25%, sterile saline solution). The digest was then filtered through fine mesh gauze, pelleted by centrifugation, washed twice in saline solution, and resuspended in a saline solution containing antibiotics (penicillin G, streptomycin and amoxicillin).

### Bioassay in mice

After declaring the persons involved in the mouse bioassay and the protocols used to the local Animal Research Ethics Committee of University of Champagne-Ardenne (URCA), 1 mL of fresh trypsic pellet was inoculated intraperitoneally into two *Toxoplasma-*free female Swiss-Webster mice (Charles River Laboratory, France). Mice were supplied with food and water *ad libitum* daily*.* In order to limit suffering and distress, mice were acclimated for 7 days after their arrival. Cages were filled with paper strips. Animal health and behavior were monitored daily. Mice were observed based on the following criteria: external physical appearance (disheveled or spiked hairs, watering eyes, bent back, tremors), behavior (exploratory behavior decrease, unusual posture, prostration), and behavioral response to external stimuli (no response). If any of these criteria were critically altered, mice were subsequently euthanized and necropsied to investigate the parasitic load. Euthanasia consisted in CO_2_ asphyxiation followed by cervical dislocation.

Mice were bled 4 weeks post-inoculation and their serum was tested for *T. gondii* antibodies with the MAT technique. Seropositive mice were euthanized 28 days post-inoculation and their brains were examined for tissue cysts.

The detailed protocol of the mouse bioassay can be found in Appendix A 6.1. of the EFSA report “Experimental studies on *T. gondii* in the main livestock species” [39].

### DNA extraction

Genomic DNA was extracted from 300 μL of digested pellets of all collected tissue samples using an EZ1^®^ DNA Tissue Kit (Qiagen, Germany), according to the manufacturer’s instructions for tissue samples.

Genomic DNA from a suspension of 2.10^5^ cultured RH-strain tachyzoites was extracted using the same protocol (EZ1^®^ DNA Tissue Kit, Qiagen, Germany). A standard curve was developed, with four 10-fold serial dilutions of the genomic DNA, corresponding to 1 × 10^0^ to 1 × 10^3^ tachyzoites/μL.

### Real-time quantitative PCR

DNA extracts from all collected tissue samples were amplified by real-time quantitative PCR (qPCR) targeting the 529 bp repeated element [27, 44]. Reactions were performed in 96-wells plates in a final volume of 25 μL on an iQ5 instrument (Biorad Laboratories GmbH, Munich, Germany). DNA oligonucleotide primers and a Taqman probe used to detect the *T. gondii* AF 487550 gene and the reaction mixture were previously described by Lélu et al. [34]. The temperature cycling conditions involved an initial incubation for 3 min at 50 °C, followed by a 3 min 30 sec incubation step at 95 °C, followed by 45 cycles of denaturation at 95 °C for 15 sec, and annealing/extension at 60 °C for 1 min. Each sample was analyzed in duplicate on the PCR plate. In total, 108 PCR reactions were performed for the naturally infected lambs (3 lambs × 9 tissues × 2 conditions: fresh/frozen × 2 PCRs: duplicate) and 312 PCR reactions for the experimentally infected lambs (3 lambs × 9 tissues × 2 conditions: fresh/frozen × 2 PCRs: duplicate + 3 lambs × 34 muscles × 2 PCRs: duplicate). A no-template control was included in every assay. The mean of the cycle threshold (Ct) value was generated during amplification from each duplicate of all collected tissue samples. All samples with a Ct-value > 40 were considered negative.

### Data analysis

#### Absolute quantification of parasites

Five μL of each serial dilution of the standard curve were used as positive controls for the qPCR. qPCR results were analyzed using CFX manager software, Version 1.6 (Bio-Rad Laboratories GmbH, Munich, Germany). The mean log-transformed starting quantity estimated from the mean Ct-values obtained for duplicates of each sample by the regression line from the dilution curve was used to determine the parasite load. The arithmetic mean log_10_-transformed DNA concentration was calculated and the detectable parasite burden was expressed as the number of parasites per gram of tissue/muscle, taking into account the weight of the sample and the volume of digested pellet.

#### Statistical analysis

We used a generalized linear mixed model to analyze the variations in the detectable parasite burden in the nine sampled tissues, according to the mode of infection (natural or experimental), and the storage conditions (fresh or frozen). The lamb and the tissue were treated as independent random effects. Two error distributions are commonly used to model count data such as detectable parasite burdens: the Poisson distribution and the negative binomial distribution. Because of the strong overdispersion observed with Poisson distributions, we chose to use negative binomial error distributions. The same statistical approach was used to model the variations of the detectable parasite burden in the 34 sampled muscles according to three characteristics: their anatomical region (head, neck, back, thorax, thoracic and pelvic limbs, abdomen and tail), their position to the trunk (cranial, dorsal, ventral, proximal, distal and caudal) and their physiologic role (postural muscles with anti-gravity and tonic activity, practically always active, versus phasic muscles supporting movement). We thus fitted three general linear mixed models of detectable parasite burden using a negative binomial error distribution, including the lamb as a random effect. As the sample muscles had all been taken from experimentally infected lambs, and stored frozen, the mode of infection and storage conditions were not included in the three statistical models. Statistical analysis was done using R software [41] and the lme4 package [2].

## Results

### Clinical observations of experimentally infected lambs

**Figure 1:**
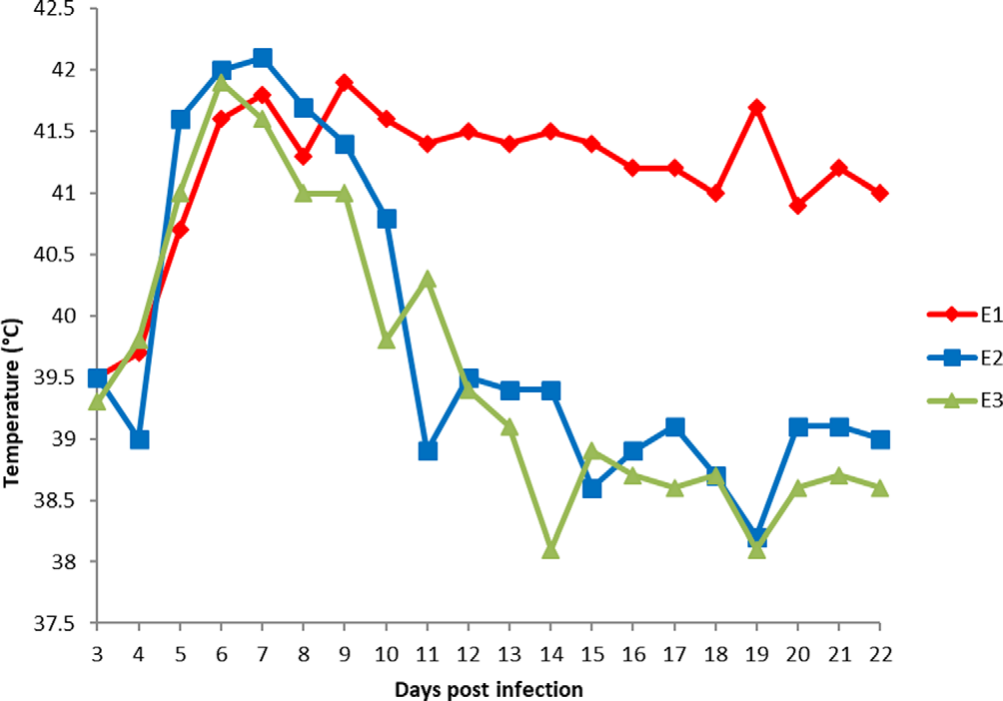
Rectal temperatures of lambs following oral infection with *Toxoplasma gondii* oocysts (3–22 dpi).

The three lambs (E1, E2 and E3) showed a febrile response, defined as a rectal temperature of 40 °C or higher, as soon as day 5 post-infection, with a maximum temperature reached on days 6 and 7 p.i. (42 °C). The rectal temperature values returned to normal on day 12 (39.4 °C) for lambs E2 and E3, while for lamb E1 the fever persisted until day 22 post-infection (Figure 1).

At the beginning of the experiment (day 3 p.i.), the three lambs each weighed 40 ± 0.5 kg, while on the day of euthanasia (days 51 and 52 p.i.), they weighted 34.6 kg, 42 kg and 47.6 kg for lamb E1, E2 and E3, respectively.

No particular clinical observations were reported for naturally infected lambs.

### Serological assays of experimentally infected lambs

**Figure 2:**
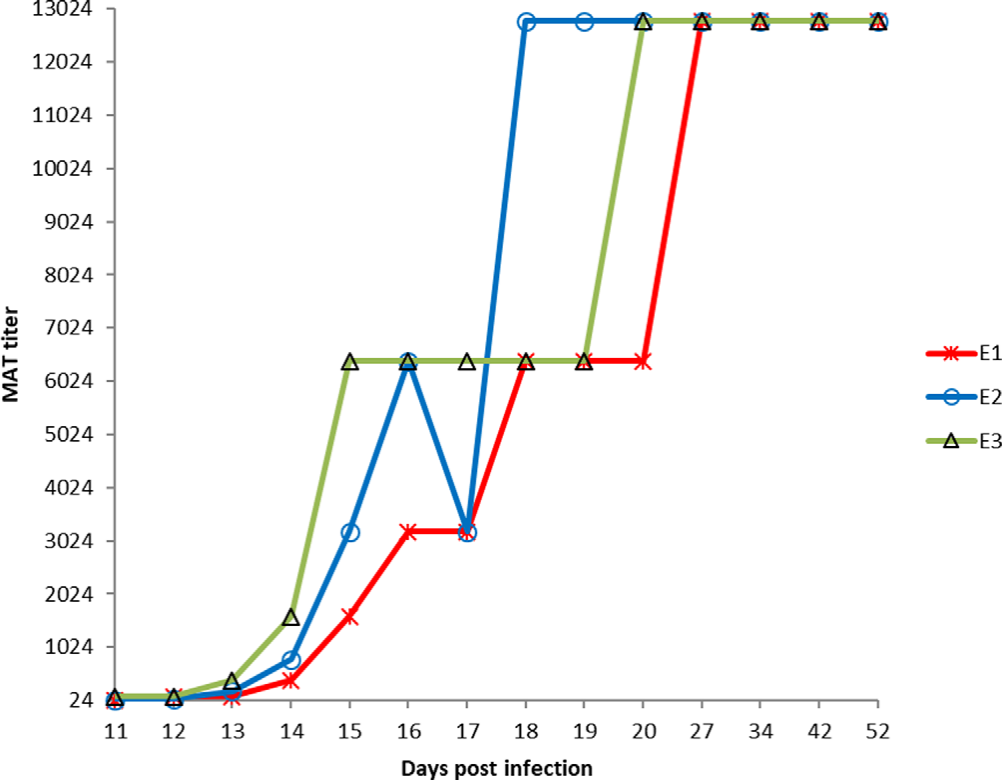
Modified agglutination test titers in experimentally infected lamb sera (11–52 dpi).

Following oral infection with 10^3^ sporulated oocysts of *Toxoplasma gondii*, specific seroconversion was detected in all three lambs, beginning with day 11 post-infection. The MAT titers on day 11 p.i. were 24 for lambs E1 and E2 respectively, and 96 for lamb E3. These titers of *Toxoplasma gondii* IgG antibodies consistently increased in the following days to reach 12,288 on day 18 post-infection for lamb E2, day 20 p.i. for lamb E3, and day 27 p.i. for lamb E1, and persisted at this titer until the end of the experiment in all three animals (Figure 2).

For the additional collected muscles (*n* = 34), MAT titers of meat juices varied from 24 to 96 (Table 1). Some meat juices were negative for lamb E1. For many muscles (*n* = 52), particularly those with presence of fascias or visible fat and connective tissue, no fluid was collected.

### Parasite detection

#### Bioassays

One hundred and eight bioassays were performed following trypsic digestion of the nine tissues (brain, heart, lung, intestine, tongue, diaphragm, quadriceps femoris muscle, intercostal muscles, and deltoid muscle samples) collected both from the experimentally and naturally infected lambs (*n* = 9 tissues × 6 lambs × 2 mice).

No *T. gondii* specific IgGs were detected by the MAT technique in mice sera inoculated with digested tissues of naturally infected lambs (N1, N2 and N3) (*n* = 54), whereas 46 mice sera inoculated with digested tissues of experimentally infected lambs (E1, E2 and E3) were positive (titer > 24). Four mice sera were negative by MAT, corresponding to the inoculation of the brain and the lung of experimentally infected lamb E3. Four mice inoculated with digested tissues of the brain and the quadriceps femoris of the experimentally infected lamb E1 died 3 weeks after inoculation and living parasites were isolated from these mice, concluding to toxoplasmosis.

#### Real-time quantitative PCR and absolute quantification of parasites

Following trypsic digestion of the nine tissues (fresh and frozen) of naturally and experimentally infected lambs, as well as the additionally 34 muscles samples (frozen) collected for the experimentally infected lambs, DNA extracts were amplified by real-time qPCR targeting the 529 bp repeated element. The detectable parasite burdens, expressed as arithmetic mean number of parasites per gram by tissue, are summarized in Tables 2 and 3 for the nine tissues and in Table 1 for the additional 34 muscles samples.

**Table 2:** Detectable parasite burden (arithmetic mean of *T. gondii* parasites per gram of tissue and range) from naturally (N) infected lambs in fresh (Fre) and frozen (Fro) digest. NR: negative results (CT value > 40).

Tissue	N1	Fre	Fro	N2	Fre	Fro	N3	Fre	Fro	Fre	Fro
	g^a^	p/g^b^		g^a^	p/g^b^		g^a^	p/g^b^		Arithmetic mean Range [min–max]	Arithmetic mean Range [min–max]
Intestine	52.00	**NR**	**NR**	59.50	0.05	**NR**	130.00	0.02	0.10	0.02 [0.00–0.05]	0.03 [0.00–0.10]
Tongue	29.00	**NR**	**NR**	35.00	**NR**	0.17	49.50	0.15	**NR**	0.05 [0.00–0.15]	0.06 [0.00–0.17]
Heart	34.50	0.47	**NR**	41.10	0.19	0.26	49.33	0.14	0.19	0.30 [0.14–0.47]	0.15 [0.00–0.26]
Quadriceps femoris	140.00	0.24	0.15	140.00	0.11	1.12	175.00	0.64	0.04	0.33 [0.11–0.64]	0.44 [0.04–1.12]
Lung	52.00	**NR**	0.16	93.77	1.11	**NR**	86.50	0.04	0.24	0.38 [0.00–1.11]	0.13 [0.00–0.24]
Intercostal muscles	82.50	1.13	0.39	93.50	0.29	0.54	125.00	0.07	0.26	0.50 [0.07–1.13]	0.40 [0.26–0.54]
Deltoid	104.00	0.10	0.19	125.00	0.04	0.37	125.00	1.92	0.56	0.69 [0.04–1.92]	0.37 [0.19–0.56]
Brain	54.25	**NR**	**NR**	51.00	2.30	1.73	51.90	**NR**	**NR**	0.77 [0.00–2.30]	0.58 [0.00–1.73]
Diaphragm	19.50	**NR**	**NR**	57.26	0.04	**NR**	48.25	6.66	0.10	2.23 [0.00–6.66]	0.03 [0.00–0.10]

a g: grams of tissue.

b p/g: calculated number of parasites per gram.

The number of *T. gondii* parasites was calculated by interpolating the average Ct values on the standard curve equivalent to 1 × 10^0^ to 1 × 10^3^ tachyzoites with tenfold serial dilutions. Estimated parasite numbers in tissue samples (detectable parasite burden) were expressed as parasite number/g lamb tissue. The range describes the lowest and highest estimated number of parasites within samples.

**Table 3:** Detectable parasite burden (arithmetic mean of *T. gondii* parasites per gram of tissue and range) from experimentally (E) infected lambs in fresh (Fre) and frozen (Fro) digest. NR: negative results (CT value > 40).

Tissue	E1	Fre	Fro	E2	Fre	Fro	E3	Fre	Fro	Fre	Fro
	g^a^	p/g^b^		g^a^	p/g^b^		g^a^	p/g^b^		Arithmetic mean	Arithmetic mean
										Range [min–max]	Range [min–max]
Lung	125.00	57.49	18.32	100.00	3.47	0.08	108.85	0.07	0.86	20.34	6.42
										[0.07–57.49]	[0.08–18.32]
Intestine	25.69	156.59	1.10	48.20	74.38	5.16	111.25	1.20	0.22	77.39	2.16
										[1.20–156.59]	[0.22–5.16]
Heart	65.15	2952.82	1307.39	60.65	724.44	181.02	60.40	195.03	49.00	1290.76	512.47
										[195.03–2952.82]	[49.00–1307.39]
Tongue	38.23	3700.57	63.80	40.80	872.63	159.30	62.00	573.86	72.92	1715.69	98.67
										[573.86–3700.57]	[63.80–159.31]
Deltoid	14.67	2715.38	78.19	49.61	2172.44	509.70	87.70	1737.06	671.91	2208.29	419.93
										[1737.06–2715.38]	[78.19–671.91]
Intercostal muscles	96.00	6845.06	202.37	141.45	692.60	349.91	179.05	423.13	258.13	2653.60	270.14
										[423.13–6845.06]	[202.37–349.91]
Diaphragm	43.80	6145.29	162.73	63.05	1346.90	91.98	53.10	763.98	123.44	2752.06	126.05
										[763.98–6145.29]	[91.98–162.73]
Quadriceps femoris	120.00	11,817.25	4185.37	133.50	1036.48	1334.73	140.60	991.93	599.01	4615.22	2039.70
										[991.93–11,817.25]	[599.01–4185.37]
Brain	53.79	26,754.09	1649.42	54.50	2243.78	86.94	55.25	18.79	46.41	9672.22	594.2
										[18.79–26,754.09]	[46.41–1649.42]

a g: grams of tissue.

b p/g: calculated number of parasites per gram.

The number of *T. gondii* parasites was calculated by interpolating the average Ct values on the standard curve equivalent to 1 × 10^0^ to 1 × 10^3^ tachyzoites with tenfold serial dilutions. Estimated parasite numbers in tissue samples (detectable parasite burden) were expressed as parasite number/g lamb tissue. The range describes the lowest and highest estimated number of parasites within samples.

For naturally infected lambs, fresh samples from heart, quadriceps femoris, intercostal and deltoid muscles were positive in all three animals, while intestine, lung and diaphragm samples were positive only in two lambs (N2, N3); brain and tongue samples in only one lamb (N2, N3 respectively) (Table 2).

For frozen samples, deltoid and intercostal muscles and quadriceps femoris were positive in all three lambs, whereas lung and heart samples were positive only in two lambs (lung of lambs N1 & N3 and heart of lambs N2 & N3); intestine, diaphragm, tongue and brain samples were positive in only one lamb (tongue and brain of lamb N2 and intestine and diaphragm of lamb N3) (Table 2).

For experimentally infected lambs, all nine tissue samples were positive for both conditions (fresh or frozen) for all three animals (E1, E2 and E3) (Table 3).

The statistical analysis showed a significant effect of the mode of infection, natural vs experimental (*p* < 0.0001), and of the detection efficiency by qPCR between fresh vs frozen digest (*p* < 0.0001). The detectable parasite burden was 3.10^−4^ times lower in samples from naturally infected lambs compared to the samples from experimentally infected lambs, while the detection efficiency of qPCR was 4.7 times higher in fresh samples than in frozen samples (Table 4).

**Table 4:** Generalized linear mixed model of the variations of detectable parasite burden in nine tissues according to the mode of infection of the lamb and to the mode of conservation of the samples (bold: significant effects, at a 0.05 threshold).

Variable	Value	Ratio of detectable parasite burden (95% CI)	*p*-value
Mode of infection	Experimental (reference)		
	**Natural**	**0.0003 (0.00006–0.002)**	**<0.0001**
Mode of conservation	Frozen (reference)		
	**Fresh**	**4.7 (2.7–8.1)**	**<0.0001**

Tissues (brain, heart, lung, intestine, tongue, diaphragm, quadriceps femoral muscle, intercostal muscles, and deltoid muscle samples) and lamb were treated as random effects.

Concerning the additional 34 muscles collected, qPCR positive results were obtained in all 3 animals for all samples, except for *Musculus digastricus* which was negative in two lambs (E1, E3) and *Musculus gluteus medius, Musculus temporalis* and *Musculus brachiocephalicus* which were negative, each in one lamb (E1, E2 and E1, respectively) (Table 1). The highest parasite estimates were observed in thoracic limbs (*Musculus supraspinatus*, *Musculus subscapularis*, *Musculus triceps brachii, Musculus infraspinatus* and *Musculus biceps brachii*) and in pelvic limbs (*Musculus biceps femoris*, *Musculus adductor, Musculus psoas major, Musculus extensor digitorum brevis, Musculus flexor digitorum superficialis, Musculus gracilis, Musculus gastrocnemius* and *Musculus semimembranosus*) (Table 1). Conversely, the detectable parasite burden was significantly lower in muscles from the head (*p* < 0.0001) (Table 5).

**Table 5:** Generalized linear mixed model of the variations of detectable parasite burden in 34 muscles according to their anatomical region, position to the trunk and physiological role (bold: significant effects, at a 0.05 threshold).

Model	Value	Ratio of detectable parasite burden (95% CI)	*p*-value
Anatomical region	Pelvic limb (reference)		
	Abdomen	0.45 (0.11–1.85)	0.27
	Back	0.56 (0.23–1.35)	0.20
	**Head**	**0.14 (0.07**–**0.30)**	**<0.0001**
	Neck	0.47 (0.17–1.32)	0.15
	Tail	0.38 (0.09–1.56)	0.18
	Thoracic limb	1.39 (0.73–2.65)	0.37
	Thorax	0.60 (0.21–1.67)	0.32
Position to the trunk	Distal (reference)		
	Caudal	0.41 (0.09–1.82)	0.24
	**Cranial**	**0.15 (0.06**–**0.37)**	**<0.0001**
	Dorsal	0.47 (0.15–1.48)	0.20
	Proximal	1.29 (0.64–2.59)	0.47
	Ventral	0.57 (0.24–1.34)	0.19
Role for physiology	Phasic (reference)		
	**Postural**	**1.91 (1.08**–**3.4)**	**0.03**

Lamb was treated as a random effect.

Taking into account the physiology of muscles, a statistically significant difference was shown in the means of the parasite loads found in postural and phasic muscles (*p* = 0.03), with a higher detectable parasite burden in postural muscles than in phasic muscles (Table 5). The lowest numbers of *T. gondii* parasites were observed in *Musculus buccinator* and *Musculus digastricus* (Table 1)*.*

### Parasite distribution in naturally and experimentally infected lambs

**Figure 3:**
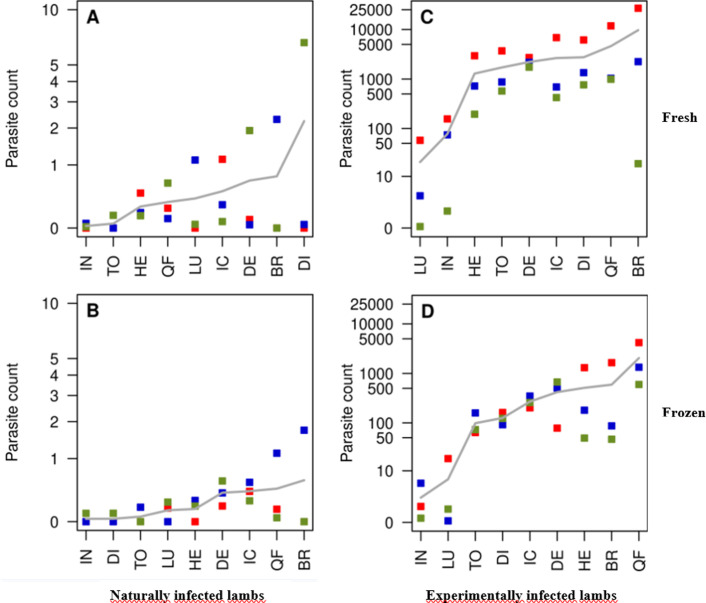
Comparison of parasite count in intestine (IN), tongue (TO), heart (HE), quadriceps femoris (QF), lung (LU), intercostal muscles (IC), deltoid (DE), brain (BR) and diaphragm (DI), in naturally (A, B) and experimentally (C, D) infected lambs, in fresh (A, C) or frozen (B, D) samples. (Parasite count is expressed as the arithmetic mean of *T. gondii* parasites per gram of tissues. Each dot represents one lamb, red dot = E1, blue dot = E2, green dot = E3; horizontal line represents the mean of the parasite count per gram of tissue for the three lambs). The number of *T. gondii* parasites was calculated by interpolating the average Ct values on the standard curve equivalent to 1 × 10^0^ to 1 × 10^3^ tachyzoites with tenfold serial dilutions. Estimated parasite numbers in tissue samples (parasite count) were expressed as parasite number/g lamb tissue.

A comparison of mean numbers of parasites per gram of tissue is presented in Figure 3 for both naturally and experimentally infected lambs for fresh and frozen samples. Among the top 3 most infected tissues, there are six samples: brain, deltoid, diaphragm, intercostal muscles, heart and quadriceps femoris, depending on the type of infection and storage conditions. Brain and quadriceps femoris are almost constantly ranked on the first two positions: brain twice in first position and twice in second position. Among the less infected tissues, in the top 3, the intestine appears to be regularly in the last position. In experimentally infected lambs, low levels of infection were found in the lungs (Figures 3C and 3D), while for naturally infected lambs, the lungs appeared to be ranked in the middle. Detectable parasite burden in the diaphragm was high in fresh samples (Figures 3A and 3C), while it was low in frozen samples (Figures 3B and 3D).

## Discussion

Following *Toxoplasma gondii* infection, the host elicits a strong, fast and specific immune response that leads the tachyzoites to find shelter in various tissues in the form of tissue cysts containing bradyzoites [14, 19]. This study focused on the detailed anatomical distribution of *T. gondii* DNA in experimentally and naturally infected lambs, with a public health perspective in mind: ranking the edible parts intended for human consumption, according to the parasite load. Still from a public health perspective, we aimed to evaluate the impact of freezing on the detection performance of the PCR assay on tissues and muscles of naturally and experimentally infected lambs, since imported meat may travel and arrive as frozen carcasses.

Concerning the clinical observations in the experimentally infected lambs (E1, E2, E3), following *T. gondii* infection, the animals developed clinical signs (fever), as soon as day 5 p.i. A febrile response around 3–5 days p.i. in sheep with *T. gondii* is a very consistent clinical finding, as described in the literature review by Dubey [19] that is compatible with acute toxoplasmosis. The fever observed in the experimentally infected lambs is a consequence of the innate immune system that boosts the effectiveness of the adaptive immune response [23]. However, for E1, the fever persisted until the end of the recording period of rectal temperatures (day 22 p.i.), possibly indicating a stronger defense response due to: a) a higher dose of parasite uptake, b) a higher detectable parasite burden, c) a weaker organism, d) the natural differences among individual clinical responses due to innate immune variations [9], or e) other infections.

Since all three lambs received a controlled oral dose of 10^3^ sporulated oocysts, with a similar clinical course observed for E2 and E3, hypotheses (a) and (d) can be ruled out to explain this prolonged fever. On the contrary, the mean number of parasites per gram of muscle, which is the highest in E1 for any of the tissues/muscles, together with the loss of weight over the period of infection (34.6 kg at the end versus 40 ± 0.5 kg at the beginning of the experiment) might explain the extended fever, reinforcing hypotheses (b) and (c), with the fast multiplication of the parasite in a weak individual.

Sheep can develop very high levels of *T. gondii* antibodies during acute infection and this level can persist for months or years [17]. In the present case, *T. gondii*-specific IgG antibodies were detected by MAT in the serum of the three experimentally infected lambs as early as day 11 p.i. at a lower dilution (1:24 for E1/E2 and 1:96 for E3), constantly increasing, especially beginning with day 14 p.i. to reach a plateau (dilution of 1:12.288) on days 18, 20 and 27 p.i. for lambs E2, E3 and E1, respectively. These results corroborate those reported by several authors [19, 21, 35, 48] who followed up the kinetics of IgG antibodies using various tests (IFA, LAT, IHA, ELISA): it has been observed an early serological response starting with day 12–14 p.i. [35, 47] with a five to eight-fold increase from baseline values in the following days to reach maximum values by day 20 p.i. or later [21]. The slight differences for the onset of seroconversion between our results and those reported in the literature might be due to the infective dose: higher infective doses induce higher antibody titers [21, 36, 39], or might be due to the serological test used, with various antigens employed to detect the antibodies: IFAT – whole tachyzoites, MAT – formalin-treated whole tachyzoites, ELISA – the P30 fraction of tachyzoites, etc.

Concerning differences in the detection of the *T. gondii*-specific antibodies, when using a different biological matrix, the meat juices of the frozen muscles, positive results were obtained for each experimentally infected lamb; however, the dilutions were much lower (1:24, 1:48, 1:96), depending on the muscles (Table 1). No antibodies were found in the meat juice of several muscles (*Musculus pterygoideus*, *Musculus brachiocephalicus, Musculus tensor fasciae latae* and *Musculus semimembranosus*) of E1, which was however the lamb with the highest detectable parasite burden. These results confirm that meat juice is not a homogenous serological matrix for lambs, as it has already been proven for naturally infected sheep or experimentally infected pigs, with a correlation between *T. gondii* titers in serum and meat juice depending on the muscle/organs [50, 52]. Moreover, postmortem changes like variations of the water-holding capacity (WHC) of muscles, that can vary greatly between animals, muscles, parts of muscles or be influenced by genetic factors, animal handling before slaughter, and meat processing could explain that for many muscles (*n* = 52), no meat juices was collected [37]. Therefore, *T. gondii* antibody detection in sheep should be performed mainly on serum, the meat juice representing an alternative biological matrix that needs to be used with caution due to the multiple disadvantages mentioned here.

Concerning the parasite detection in the fresh and frozen tissues of infected lambs, *T. gondii* DNA was detected in each of the three animals of the two groups for both storage conditions, but not at the same concentration. We observed a statistically significant (*p* < 0.0001) higher concentration of *T. gondii* DNA in the fresh tissues of naturally and experimentally infected lambs than in the frozen tissues of naturally and experimentally infected lambs: the amount of *T. gondii* DNA was 4.7 times higher in the fresh digests than in the frozen digests of the infected lambs. Freezing and long-term storage at −20 °C resulted in a loss of ability to detect specific parasite DNA in tissues. As we performed digestion, extraction and quantitative PCR on these samples after their storage at −20 °C, our findings suggest short-term stability of *T. gondii* DNA in unprocessed frozen samples involving a lower DNA efficiency detection by PCR. A combined action of nucleases and proteases may have degraded the DNA in the unprocessed samples, whereas DNA is stable for long-term conservation in appropriate buffer once it has been extracted. Similarly, James et al. [29] showed that the sensitivity of PCR was reduced by storage at +4 °C or −20 °C for 48 h of a cell culture tachyzoite suspension, while Delhaes et al. [12] showed that long-term storage at −20 °C of extracted DNA from amniotic fluids did not affect PCR sensitivity. Furthermore, Hasan et al. [25] showed a temperature-dependent loss of pathogen-specific DNA (cytomegalovirus) or RNA (enterovirus) sensitivity by real-time PCR in unprocessed clinical samples (serum and cerebrospinal fluids, respectively).

In conclusion, for molecular detection of *T. gondii* infection, optimal and appropriate methods must be applied to preserve *T. gondii* DNA, such as performing DNA extraction promptly after sampling when *T. gondii*-PCR is delayed. The significant effect of the conservation mode of biological samples should be carefully evaluated when *T. gondii* presence is being investigated in long-term frozen samples.

When comparing naturally versus experimentally infected lambs, we noticed that *T. gondii* DNA was present in each of the nine tissues of the experimentally infected lambs, while *T. gondii* DNA was not detected in several tissues (diaphragm, intestine, lung, brain, heart and tongue) of naturally infected lambs. This result might be explained by the variability in the detectable parasite burden since the statistical analysis revealed a significantly (*p* < 0.0001) higher detectable parasite burden in tissues collected from experimentally infected lambs than in tissues collected from naturally infected lambs: the detectable parasite burden was 3.10^−4^ times less in tissues from naturally infected lambs compared to the tissues from experimentally infected lambs. Several studies [15, 21, 22] reported that a 10^1^ to 10^3^-fold difference in oocyst infection dose influenced the frequency of detection of *T. gondii* in ovine tissues; our results also suggest that the detectable parasite burden depends on the infection dose/mode. Presumably the naturally infected lambs had a significantly lower infection dose. Another factor that could account for these differences in DNA detection is the breed: our study was conducted on two different breeds, Pré Alpes du Sud for the experimentally infected lambs, and Vendéen for the naturally infected lambs, while it has been suggested that certain lines or breeds have a particular genetic susceptibility to *T. gondii* [8]*.*

The same difference between experimentally and naturally *T. gondii* infected lambs was noticed for the bioassay: no *T. gondii* specific IgGs were detected in any of the 54 mice inoculated with the tissues of naturally infected lambs, while 50 mice sera, inoculated with the tissues of experimentally infected lambs, were positive. A significant correlation between increasing MAT titers and the probability of isolating live parasites was observed in naturally infected sheep [50]: when the titer, on cardiac fluid, was higher than 16, parasites were isolated in 65% of the cases, when it was lower than 16, isolation failed in 95% of the cases. Considering that by the age of 3-months, maternally derived colostral antibodies are no longer detectable [51], it suggests that the 24 MAT titer, on sera, used for the selection of our naturally infected lambs lacks sensitivity for parasite isolation, confirming *in fine* a significantly lower detectable parasite burden for this type of infection.

Regarding the four negative mice inoculated with tissues of experimentally infected lambs, they correspond to the inoculation of brain and lungs of the same lamb, E3. These results can be explained by: a) a lower detectable parasite burden of these tissues; b) a lack of mouse-bioassay sensitivity; c) a lack of MAT sensitivity, and d) a technical problem.

Concerning hypothesis (a), Table 3 shows that the detectable parasite burden is significantly lower in brain and lungs of lamb E3 compared to the same organs for lambs E1 and E2, respectively: 18.79 versus 26,754.09 and 2243.78 parasites/g; 0.07 versus 57.49 and 3.47 parasites/g. Since qPCR performed on the same tissues (brain and lungs) gave positive results, a lack of mouse-bioassay sensitivity is incriminated (hypothesis b). Moreover, Da Silva et al. [11] have already shown that PCR performs better than mouse bioassays in acid pepsin digest of brain, lung and muscle tissues of sheep from Brazilian slaughterhouses. The cut-off chosen for the MAT analysis of sera of inoculated mice, might have been too high, justifying hypothesis (c), suggesting that a qPCR performed on the mouse brain would have been more informative, like reported by Opsteegh et al. [40]. Most probably, this negative mouse bioassay can be explained by the last hypothesis (d), with an insufficient volume of inoculum, combined with a low detectable parasite burden: from 55.25 g of brain and 108.85 g of lungs, corresponding to 10–15 mL of digest, only 1 mL/mouse were inoculated into two mice.

Concerning the distribution of *T. gondii* among the nine tissues according to the mean number of parasites per gram of meat, a specific pattern was observed, in both naturally and experimentally infected animals, for the two storage conditions (fresh or frozen). In the top 2 most infected tissues, there are mainly two samples: brain and quadriceps femoris, while the 2 consistently least infected tissues were intestine together with either lung, tongue or diaphragm. The rest of the tissues have a non-specific distribution pattern, varying according to the type of infection and storage condition. The brain, a major predilection site for *T. gondii*, since the parasite can escape cell-mediated immunity, was also found on top of the list in a recent literature review [38] for several species: sheep, pigs and goats, together with the heart. Discordantly, in our study the heart was ranked in the lower half of the list, the place in the tandem being substituted by the quadriceps femoris. This might be explained by the absence of quadriceps femoris’ investigation in the reviewed studies; however, the low rank of heart might also be explained by the low number of animals that we analyzed so far. The low parasite load detected in the intestine is in accordance with the results described by Verhelst et al. [49] who found a decrease of the parasite DNA levels in the intestine during the acute phase of *T. gondii* infection in sheep to become undetectable 3 weeks p.i. Similar results have been found by Opsteegh et al. [38] when detecting *T. gondii* in ovine tissues, with the intestine on the position right after the lungs and not far away from the tongue. Similarly, Dubey and Frenkel [14] have shown a tissue development specificity in mice inoculated with *T. gondii* tachyzoites: cysts developed slower and less frequently in lungs, liver, spleen and kidneys than in the brain and in cardiac and skeletal muscles.

Concerning the distribution of *T. gondii* among the 34 additionally collected muscles according to the number of parasites per gram of meat, a predilection of the parasite for the thoracic and pelvic limb muscles, as well as for the proximal and postural muscles versus distal and phasic muscles was observed.

No significant difference (*p* > 0.05) was observed in the parasite load between thoracic and pelvic limb muscles (*p* = 0.37). Opsteegh et al. [38] ranked nonetheless pelvic limb muscles a few places higher than thoracic limb muscles. However, the authors based their ranking on only three articles and with no precise anatomical specification of the tested muscles. Therefore, one should keep in mind that front or hind limb muscles present a similar detectable parasite burden, in accordance with the similar physiological role played by these muscles. Regarding the parasite load between proximal and distal muscles, no significant difference was found (*p* = 0.47). However, a high detectable parasite burden was observed in the proximal muscles compared to the distal muscles. It is plausible that this difference may be due to the blood vessels and their diameter: for the proximal muscles, the size of blood vessels is significantly higher than for the distal muscles, involving a higher quantity of circulating blood, possibly transporting a higher number of parasites to these muscles. This explanation is consistent with the findings of Dellacasa-Lindberg et al. [13] who suggested that vascularization could influence the distribution pattern of *T. gondii* in various areas of the mouse brain. On the other hand, Ranjbar et al. [43] showed that the pattern of vascularization in response to exercise training is different between fast- and slow-twitch muscles. This difference in vascularization may explain the difference in detectable parasite burden between postural and phasic muscles and their detailed anatomical architecture: postural muscles contain mainly slow-twitch muscle fibers; they are tight and short and are involved in maintaining posture and supporting endurance. Phasic muscles are mostly fast-twitch muscles: they support movement and fine motor skills but tire and stretch more easily than postural muscles. At the same time, the postural muscles are practically always active and have a greater capacity for sustained work compared to phasic muscles which tend to be weak. This major difference in the physiological role may give a second explanation for this variation in detectable parasite burden. Hughes and Harley [28] developed the same hypothesis to explain why the nematode *Trichinella spiralis* is attracted by the more active skeletal muscle when compared to less active skeletal muscle (resting muscle) in response to a microenvironmental stimulus (mainly electrical stimuli). In our study, a significant difference (*p* = 0.03) was observed in the detectable parasite burden among postural and phasic muscles, with parasite loads in postural muscles 1.91 times higher than in phasic muscles.

However, in our study, low numbers of *T. gondii* parasites reached the *Musculus buccinator* and *Musculus digastricus* which are still very active muscles in ruminants. This surprising result might be due to a technical problem (low quantity of muscle, loss of PCR sensitivity, etc.) or could indicate that the parasite is either cleared or is leaving these muscles before 8 weeks p.i. The detectable parasite burden was significantly lower in cranial and head muscles (*p* < 0.0001) by comparison to the proximal and distal muscles on the one hand, and thoracic and pelvic limb muscles on the other. These results need to be confirmed by sampling more animals.

In conclusion, our results have shown that virtually all studied pieces of lamb harbor viable *Toxoplasma gondii* tissue cysts or *T. gondii* DNA. High levels of parasite DNA were observed in skeletal muscles and more particularly in edible portions such as quadriceps femoris muscle, intercostal muscles, deltoid muscle, diaphragm and thoracic and pelvic limb with a significant difference in detectable parasite burden between fresh and frozen samples or natural and experimental infection. Eating undercooked lamb meat (*agneau rosé*) poses a risk to public health and therefore, lamb meat should be thoroughly cooked or frozen before consumption [31]. To our knowledge, this is the first study that presents highly detailed information on the anatomical distribution of *T. gondii* DNA in naturally and experimentally infected lambs using fresh and frozen samples. Further investigations need to be done in order to confirm the above-mentioned differences in more animals and in different breeds.

## Conflict of interest

All individual authors declare that they have no conflict of interest (financial, personal or other).
